# Comparative evaluation of four rapid diagnostic tests that detect human *Trypanosoma cruzi-*specific antibodies to support diagnosis of Chagas Disease in urban population of Argentina

**DOI:** 10.1371/journal.pntd.0011997

**Published:** 2024-03-15

**Authors:** Rocío Rivero, M. Soledad Santini, Constanza Lopez-Albizu, Marcelo Rodriguez, Adriana Calbosa, Daniela Oliveto, Mónica Esteva, Margarita Bisio, Laura C. Bohorquez

**Affiliations:** 1 ANLIS Administración Nacional de Laboratorios y Salud, Instituto Nacional de Parasitología “Dr. Mario Fatala Chaben”, Buenos Aires, Argentina; 2 CONICET Consejo Nacional de Investigaciones Científicas y Técnicas, Buenos Aires, Argentina; 3 FIND, Foundation for Innovative New Diagnostics, Geneva, Switzerland; Wadsworth Center, UNITED STATES

## Abstract

**Background:**

Chagas disease (CD), caused by the parasite *Trypanosoma cruzi*, is the most important endemic anthropozoonosis in Argentina. Since 2010, the World Health Organization has highlighted the urgent need to validate diagnostic systems that allow rapid detection of *T*. *cruzi*, infection in primary healthcare centers. Serological rapid diagnostic tests (RDTs) for *T*. *cruzi*, infection could be used to improve case management, as RDTs do not require specialized laboratories or highly trained staff to use them. We aimed to generate unbiased performance data of RDTs in Argentina, to evaluate their usefulness for improving *T*. *cruzi*, diagnosis rates.

**Methods and principal findings:**

This is a retrospective, laboratory-based, diagnostic evaluation study to estimate the clinical sensitivity/specificity of four commercially available RDTs for *T*. *cruzi*, using the Chagas disease diagnostic algorithm currently used in Argentina as the reference standard. In total, 400 serum samples were tested, 200 from individuals with chronic *T*. *cruzi* infection and 200 from individuals not infected with *T*. *cruzi*. All results were registered as the agreement of at least two operators who were blinded to the reference standard results. The sensitivity estimates ranged from 92.5–100% (95% confidence interval (CI) lower bound 87.9–98.2%); for specificity, the range was 76–96% (95% CI lower bound 69.5–92.3%). Most RDTs evaluated showed performances comparable with the reference standard method, showing almost perfect concordance (*Kappa* 0.76–0.92).

**Conclusions:**

Our study demonstrates that, under controlled laboratory conditions, commercially available RDTs for CD have a performance comparable to the Argentinian diagnostic algorithm, which is based on laboratory-based serological tests. For the next stage of our work, the RDTs will be evaluated in real-world settings.

## Author summary

*Trypanosoma cruzi* is the causative agent of Chagas disease, the most significant transmissible disease between animals and humans in Argentina. It is estimated that two-thirds of individuals with the disease currently reside in urban areas, and globally, only 10% of those affected are aware of their condition. The diagnosis of chronic infection requires conducting at least two different laboratory tests mainly available at reference laboratories, which poses logistical and economic challenges for both the patients and the health systems, and it often results in limited access to high-quality diagnosis. Primary healthcare has the potential to address around 90% of the population’s healthcare needs over their lifetime. Although there are rapid diagnostic tests for *T*. *cruzi* infection, that could be potentially incorporated at lower levels of the healthcare system in Argentina, there is a lack of independent evaluation of the available tests. In fact, since 2010 the World Health Organization emphasized the need of evaluating diagnostic tests that enable the rapid detection of *T*. *cruzi* infection in primary care centers. In this study, we assessed under controlled laboratory conditions the ability of the rapid diagnostic tests for confirming or ruling out the chronic infection in patients in Argentina.

## Introduction

Chagas disease (CD) is endemic in 21 continental American countries. The migratory movement of people has increased in recent decades due to push factors, such as poverty and unemployment, and pull factors, including better employment conditions and family reunification [1]. As a result, CD is no longer exclusively a rural problem specific to Latin America [2–4].

The World Health Organization (WHO) estimates that 70 million people worldwide are at risk of being infected by *Trypanosoma cruzi*, the parasite that causes CD. Furthermore, it is estimated that 6 million people are infected with *T*. *cruzi*, with two thirds of these individuals currently living in urban areas; however, just 10% of these infected individuals have been diagnosed, preventing them from receiving appropriate care [5–7].

According to the WHO (2010), it is estimated that there are 1,350,000 individuals infected with *T*. *cruzi* in Argentina. The Argentinian 2011–2016 National Strategic Plan for Chagas disease aimed (a) to interrupt the transmission of *T*. *cruzi* and (b) to reduce the morbidity and mortality due to CD and the subsequent socioeconomic impact [8].

The discussion of CD as a public health issue should not be limited simply to estimates of the number of infected individuals and reported numbers of cases. Individuals living in vulnerable situations or who are members of marginalized populations are often more susceptible to *T*. *cruzi* infection and associated morbidity, due to a variety of factors including a widespread lack of access to formal education, timely healthcare, and adequate living conditions. The reasons for such deprivation are complicated, but ultimately result in the persistence of inequalities in communities affected by CD in Latin America [9].

Pharmacologic treatment in its acute stage of CD can cure the disease and prevent progression and disrupts the transmission cycle in the case of future infected pregnant women [10]. However, treatment exhibits reduced efficacy during the chronic stage of the disease and requires a long period of administration, which frequently results in undesirable adverse reactions to the drugs used [11].

Currently, access to diagnosis is the main barrier to receiving appropriate treatment for CD. In the absence of a gold standard, the diagnosis of chronic CD is a complex process, involving an algorithm that includes at least two laboratory determinations. According to the Pan American Health Organization (PAHO), the diagnostic standard for patients with suspected chronic CD is the combination of two serological tests that are based on different principles and antigens e.g., an enzyme-linked immunosorbent assay (ELISA), indirect hemagglutination (IHA), or indirect immunofluorescence (IIF), with a third serological test if the results are discordant [12,13].

A healthcare system that offers timely access to reliable diagnosis is the basis for the implementation of policies that guarantee access to appropriate treatment [12]. As long ago as 2010, WHO proposed the need to validate diagnostic systems in primary healthcare centers that allow the rapid detection of *T*. *cruzi* infection as a strategy to reduce morbimortality due to CD [14]. Since then, some studies have been carried out to evaluate the performance of lateral flow assays, also known as rapid diagnostic tests (RDTs), in different population groups and with different sample types. The advantages of RDTs compared with the diagnostic standard proposed by PAHO include that RDTs do not require highly skilled or trained operators to use them, do not require refrigeration of the reagents used, have a short time from sampling to results, and allow the use of different sample types; these features mean that RDTs are also useful as field screening assays [15].

Recent field and laboratory studies, as well as systematic reviews, have found that RDTs demonstrate very good sensitivity and specificity (with 95% confidence interval (CI) ranges from 95–100%), with RDTs being proposed as an alternative to the current diagnostic standards in some regions [16–20]. However, a high risk of bias was detected in some publications in relation to the design of clinical trials and reference standards used [20]. Furthermore, the diagnostic performances of RDTs for CD have shown high variability, with sensitivity values ranging from 33 to 100% and specificity values ranging from 94 to 99.9% [13,21]. It is necessary to evaluate commercially available RDTs in various epidemiological scenarios and in different clinical settings where medical care for CD is provided. However, before conducting prospective trials, it is necessary to establish the performance of the tests under controlled laboratory conditions.

The primary objective of this study was to evaluate, under controlled laboratory conditions, the sensitivity and specificity of four commercially available *T*. *cruzi* RDTs, using the current CD diagnostic algorithm as a reference standard method and following the guidelines of both the Argentina’s Ministry of health and PAHO/WHO. As a secondary objective, we evaluated the agreement between the results of RDTs and the reference standard.

## Materials and methods

### Ethical considerations

Human samples were obtained in accordance with the general ethical principles outlined in the Declaration of Helsinki and the International Committee for Harmonization guidelines for Good Clinical Practice; the samples were handled according to Good Clinical Laboratory Practice. The study protocol (NTD008) was approved by the Ethics and Research Committee of INP (authorization number 6, Buenos Aires, 26 de April de 2022/RENIS CE000218). All procedures were performed on samples from individuals who had previously obtained a medical order of diagnosis as part of the routine standard of care and who had provided informed written consent for their additional or archived/remnant samples to be used for research purposes at the study site.

### Study design and participants

This study was performed at the Instituto Nacional de Parasitología “Dr. Mario Fatala Chaben” (INP) in Administración Nacional de Laboratorios y Salud (ANLIS), Buenos Aires, Argentina. INP is the national reference center for the diagnosis of CD in the country, where patients are referred with the prior medical order from the public health system of Argentina or from national blood banks, or self-motivated. This was a retrospective, laboratory-based evaluation study of commercially available *T*. *cruzi* RDTs that use serum samples. There were a total of 5,929 individuals registered in the data base with serological results for CD, who attended INP from January 3 to December 23, 2019.

### Potentially eligible participants

In order to surface the minimum sample size, 468 samples (from the 5,929 individuals registered in the data base of the study site) were selected by convenience sampling, *i*.*e*. accessible samples at the moment of conducting the study.

### Eligibility criteria

Remnant serum samples from Argentine patients aged more than 18 years, stored at -20°C ± 5°C, and with diagnosis reference standard test results for chronic CD included. Samples were excluded if there was evidence of inappropriate storage, the presence of microbial or fungal contamination, or the presence of chemical contamination, e.g., solvents or reagents.

### Sample size

The sample size was determined to provide reasonable confidence and precision to estimate the performance of each test under evaluation for the detection of antibodies against *T*. *cruzi* [22]. We based our calculations on sensitivity/specificity estimates ranging from 85 to 97.5%. A total of 151 positive (respectively negative) samples ensured an estimation of sensitivity (respectively specificity) within the above range, with the following precision: 85% ± 5.8, with an alpha error of 0.05 (95% confidence level) to describe performance (S1 Table). Considering 30% of potentially poor-quality specimens a total of 200 positive and 200 negative samples (for chronic *T*. *cruzi* infection), were used in order to surface the minimum sample size.

### Test methods

#### Index tests

All RDT operators were blinded to the clinical or laboratory characterization of the samples tested and the result interpretation of each other. The RDTs used in the study were selected from commercially available RDTs that detect immunoglobulin G (IgG) antibodies specific to recombinant *T*. *cruzi* antigen based on the following criteria: (a) the RDT is registered for use in Argentina, complies with the registration in the national regulatory agency, Administración Nacional de Medicamentos, Alimentos y Tecnología Médica (ANMAT), (b) the RDT is registered and used in other CD-endemic countries, and (c) the RDT could be procured from the manufacturer/local distributor in Argentina. Initially, seven RDTs were to be included in the study; however, two of them, including Chagas Strep and Chagas Detect Plus Rapid Test (InBios, Inc, USA) were discontinued for sale at the time the study was conducted (this was confirmed by the manufacturer); a third test, the OnSite Chagas Ab Rapid test/Chagas Ab Combo Rapid Test CE (CTK Biotech, USA) was not registered in Argentina, and the product for research-use only could not be procured by the local distributor. Thus, the products that were not available in Argentina at the time the study was conducted were not included. In total, four RDTs were evaluated: WL Check Chagas by Wiener Lab (Argentina), SD Chagas Ab Rapid by Standard Diagnostic (Korea), Chagas Rapid First Response by Lemos Laboratories (Argentina), and ACCU-TELL Chagas Cassette by AccuBiotech Co. Ltd (China).

To perform each index test, information regarding its operating and storage requirements, as well as the manufacturing details, were obtained from the instructions for use (IFU) provided by the manufacturer (Table 1). All index test results obtained at the study site were for research use only; they were not used for diagnosis or to make decisions with regards to treatment. Each sample was divided into four aliquots, and each aliquot was independently and blindly tested using each index test, with the results independently interpreted by two operators and then recorded. A third staff member had the deciding vote in cases where there was discordant interpretation, i.e., the final consensus result was the agreement of at least two blinded operators.

**Table 1:** Manufacturer’s details for the rapid diagnostic tests evaluated.

Test name	Test abbreviation	Manufacturer (country)	Sanitary registration in Argentina (ANMAT)	Sample volume (μl)	Buffer volume (number of drops)	Interpreting time (minutes)	Target
WL Check Chagas	WL	Wiener Lab (Argentina)	Yes	40	3	25–35	Specific recombinant antigens from epimastigote and trypomastigote stages of *T*. *cruzi*
SD Chagas Ab Rapid	SD	Standard Diagnostic (Korea)	Yes	100	NA	15	H49, 1F8
Chagas Rapid First Response	FR	Lemos Laboratories (Argentina)	Yes	5	2	20–30	Recombinant *T*. *cruzi* antigens (not specified)
ACCU-TELL (Chagas Cassette)	Accu	AccuBiotech Co., Ltd (China)	No (RUO products were used)	15	2	< 15	Not reported

(ANMAT) Administración Nacional de Medicamentos, Alimentos y Tecnología; (NA) not applicable; (RUO) research-use only

#### Reference standard

All samples tested were previously characterized as positive or negative for *T*. *cruzi* at the study site, using PAHO and national guidelines (agreement of at least two serological tests, including ELISA, IHA, or IIF) as the reference standard. In-house tests were developed previously at INP, using the following antigens: i) ELISA: lysate of epimastigotes of *T*. *cruzi* strain Tul2, ii) IHA: lysate of epimastigotes of 29 *T*. *cruzi* strains, and iii) IIF: whole epimastigotes of *T*. *cruzi* strain Tul2 preserved in formaldehyde. The tests were performed following standard operating procedures used at INP [21] (S1 Data contains the reference test results).

#### Data management

The data generated during the study were first recorded on paper forms endorsed by the INP Quality Assurance System and then transferred to an electronic database (OpenClinica Enterprise, 4.0). Standardized, high-quality photographs of the RDTs results were taken by each operator, using a smartphone-based app (TiraSpot, developed by SpotLab, Spain). The anonymized RDTs images collected were securely stored on the TeleSpot cloud platform (SpotLab, Spain), from where a single database containing all of the photographs was created [23].

The data, including the images stored on the TeleSpot cloud and the results stored in Open Clinica Enterprise, were verified by the local monitor to ensure their accuracy, correctness, and consistency with the original documents, in 5% of the critical fields of the reported data, considering a maximum acceptable error of 2%.

#### Quality control

To corroborate the ability of the RDTs to detect antibodies against the diverse range of *T*. *cruzi* strains, each index test was challenged with the WHO anti-*Trypanosoma cruzi* I and II Antibody Reference Panel (NIBSC), prior to the start of the study (S1 Fig).

#### Statistical analysis

A statistical analysis plan was developed prior to the initiation of the study. The parameters to be estimated included sensitivity, specificity, likelihood ratios, and the false-positive and false-negative rates of the index test in comparison with the positivity or negativity of *T*. *cruzi* infection of the sample defined with the reference standard method. Point estimates were reported with 95% confidence intervals (95% CI) using the Wilson scoring method. The Cohen’s *Kappa* score was determined to indicate the agreement between index tests and reference test.

After the initial results were seen, a Z-score test was used to detect statistically significant *Kappa* index. In addition, a three-way evaluation was also carried out (as recommended by the Clinical and Laboratory Standards Institute), Z-score test was also used to compare the diagnostic performance between pairs of methods and thus assess whether there were significant differences between the methods compared with the reference diagnostic algorithm [24].

#### Usability

A usability score for each index test was determined through the use of previously published standardized questionnaires [18], using qualitative variables related to the ease of use and interpretation of the results, and the subjective opinion of the operators, which were completed by each of the four highly skilled laboratory technicians belonging to the study team.

## Results

**Fig 1:**
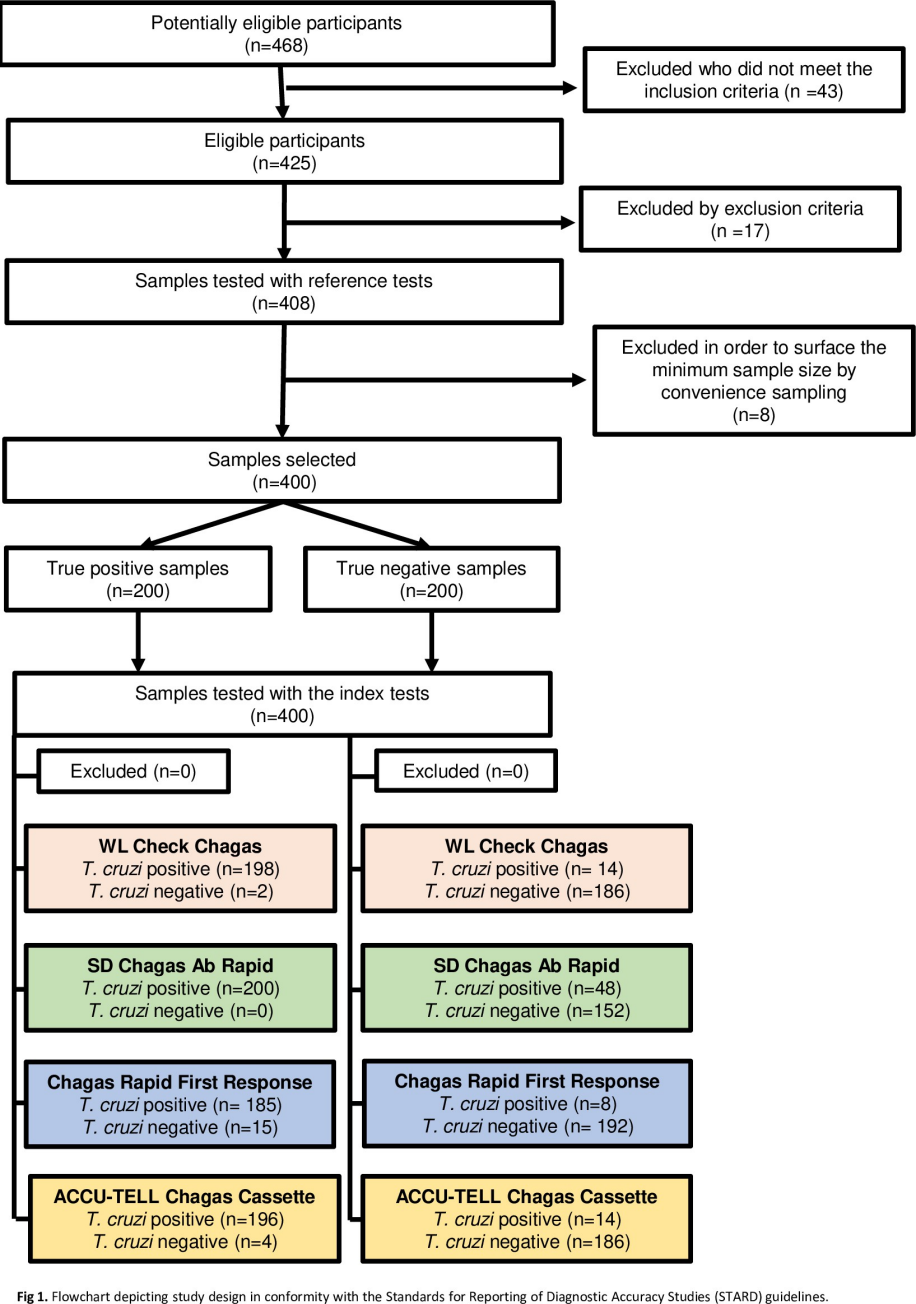
Flowchart depicting study design in conformity with the Standards for Reporting of Diagnostic Accuracy Studies (STARD) guidelines.

A flowchart depicting the study design and flow of participants samples, in conformity with the Standards for Reporting of Diagnostic Accuracy Studies (STARD) guidelines [25] is shown in Fig 1.

### Study population

In 2019, 5,929 individuals attended INP, in Buenos Aires, Argentina, to perform *T*. *cruzi* serology. Considering the eligibility, the first 200 negative samples and the first 200 positive samples with a volume greater than 500 μl, in good preservation condition, and with available reference standard results from INP, were conveniently selected (Fig 1). Regarding the 400 samples, the median age of the individuals was 49 years (range 18–84). The place of birth of these individuals was Argentina (253/400, 63.2%), Bolivia (99/400, 24.7%), Paraguay (24/400, 6%), Uruguay (2/400, 0.5%), Peru (1/400, 0.2%), and Venezuela (1/400, 0.2%); place of birth data was not available for 20/400 individuals (5%). All individuals whose samples were included had permanent residence in Argentina and were therefore considered to be users of the national health system.

### Quality control

During the process of verifying the recorded data, an acceptable error of 1.7%, in 5% of the critical fields, was obtained, including the images stored on the TeleSpot cloud platform and the results reported in OpenClinica Enterprise, compared with the original documents.

The capacity of the four index tests to detect antibodies anti-*T*. *cruzi* was evaluated with a serological external quality assurance panel. All index tests were able to detect anti-*T*. *cruzi* antibodies in human sera samples from regions where DTUs TcI and TcII are endemic, i.e., Mexico and Brazil respectively.

### Evaluation of RDTs

Table 2 shows true-positive, false-negative, true-negative and false-positive results and the estimated statistical parameters for the four RDTs evaluated. The SD Chagas Ab Rapid (Standard Diagnostic, Korea) showed the highest sensitivity (100%, 95%CI: 98.2–100%), followed by WL Check Chagas (Wiener Lab, Argentina) (99%, 95%CI: 96.4–99.9%), and Accu-tell (98%, 95%CI 95.0–99.5%). The Chagas Rapid First Response (Lemos Laboratories, Argentina) showed lowest sensitivity (92.5%, 95%CI: 87.9–95.7%). The Chagas Rapid First Response (Lemos Laboratories, Argentina) showed highest specificity (96%, 95%CI: 92.3–98.3%) while SD Chagas Ab Rapid (Standard Diagnostic, Korea) had the lowest specificity (76%, 95%CI: 69.5–81.7%).

**Table 2:** Estimated performance for all index tests (RDTs).

Test name	Sensitivity (95% CI)	Specificity (95% CI)	*Kappa* index (95% CI)	Classification by *Kappa* value	False-positive and false-negative				Z-score		Likelihood ratio	
					Positive test (CI 95%)	Negative test (Miettinen-Nurminen CI 95%)
							True-positive	False-negative	True-negative	False-positive	Z-statistic	p-value
WL Check Chagas	99% (96.4% - 99.9%)	93% (88.5% - 96.1%)	0.92	Almost perfect	198	2	186	14	6.13	1	14 (8.68–23.49)	0.01 (0.00–0.04)
			(0.88–0.96)									
SD Chagas Ab Rapid	100% (98.2% - 100%)	76% (69.5% - 81.7%)	0.76	Substantial	200	0	152	48	-1.27	0.10	4.1 (3.29–5.37)	0.0 (0.00–0.02)
			(0.70–0.82)									
Chagas Rapid First Response	92.5% (87.9% - 95.7%)	96% (92.3% - 98.3%)	0.89 (0.84–0.93)	Almost perfect	185	15	192	8	3.65	0.99	23.1 (12.01–45.38)	0.02 (0.05–0.12)
ACCU-TELL (Chagas Cassette)	98% (95.0% - 99.4%)	93% (88.5% - 96.1%)	0.91	Almost perfect	196	4	186	14	5.31	1	14 (8.59–23.26)	0.07 (0.01–0.05)
			(0.87–0.95)									

^1^ Do not reject the null hypothesis (H0: κ ≥ 0.8), the agreement beyond chance is greater than or equal to 0.8 at the 5% significance level.

No invalid tests were reported from 3/4 RDT, only WL Check Chagas (Wiener Lab, Argentina) had one invalid test result this sample was repeated.

Table 2 shows the *Kappa* index for overall agreement between the results of each RDT and the results of the reference standard, with 95% CI. The level of concordance with the reference standard of three of the four RDTs was 0.89 to 0.92 (for the WL Check Chagas by Wiener Lab, Argentina, Chagas Rapid First Response by Lemos Laboratories, Argentina, and ACCU-TELL Chagas Cassette by AccuBiotech Co. Ltd, China), i.e., an almost perfect concordance. The SD Chagas Ab Rapid by Standard Diagnostic, Korea (κ = 0.76) indicated a substantial agreement. There were no statistically significant differences in the kappa index for any of the RDTs (Table 2, overall agreement between each RDT and the reference standard).

The likelihood ratios were highly relevant for the WL Check Chagas (Wiener Lab, Argentina) and ACCU-TELL Chagas Cassette (AccuBiotech Co. Ltd, China) and Chagas Rapid First Response (Lemos Laboratories, Argentina (Table 2). Positive diagnoses results were missed by the following proportions for each test: WL Check Chagas (Wiener Lab, Argentina), 1%; SD Chagas Ab Rapid (Standard Diagnostic, Korea), 0%, Chagas Rapid First Response (Lemos Laboratories, Argentina) 7.5%; and ACCU-TELL Chagas Cassette (AccuBiotech Co. Ltd, China), 2%.

The three-way analysis showed that, in terms of sensitivity, all pairs of tests were statistically significantly different to each other, except for the WL Check Chagas (Wiener Lab, Argentina) compared with the SD Chagas Ab Rapid (Standard Diagnostic, Korea) (p = 0.1573); and the WL Check Chagas (Wiener Lab, Argentina) compared with the ACCU-TELL Chagas Cassette (AccuBiotech Co. Ltd, China) (p = 0.4142) (Table 3).

**Table 3:** Three-way analysis (significant differences in sensitivity and specificity between two methods, in a paired fashion versus the reference algorithm).

Comparison	Parameter	Proportion difference	Newcombe	Z-score		Significant difference
			95% CI	Z-statistic	p-value	
**WL vs. SD**	Sensitivity	-0.01	-0.03–0.01	-1.41	0.1573	No
	Specificity	0.17	0.11–0.22	5.83	<0.0001*	Yes
**WL vs. Accu**	Sensitivity	-0.01	-0.04–0.01	-0.82	0.4142	No
	Specificity	0.00	-0.01–0.01	-	-	No
**WL vs. FR**	Sensitivity	0.06	0.02–0.11	3.15	0.0016*	Yes
	Specificity	-0.03	-0.07–0.01	-1.28	0.2008	No
**FR vs. Accu**	Sensitivity	-0.05	-0.10–0.01	-2.84	0.0045*	Yes
	Specificity	0.03	-0.01–0.07	1.28	0.2008	No
**SD vs. FR**	Sensitivity	0.07	0.04–0.12	3.87	0.0001*	Yes
	Specificity	-0.2	-0.26–0.13	-5.35	<0.0001*	Yes
**SD vs. Accu**	Sensitivity	0.02	-0.002–0.05	2.00	0.0455*	Yes
	Specificity	-0.17	-0.22–0.11	-5.83	<0.0001*	Yes

*Significant differences in sensitivity and specificity between two methods, in a paired versus the reference algorithm. The asterisks indicate p values < 0.05.

In terms of specificity, all pairs of tests were statistically significantly different except for the WL Check Chagas (Wiener Lab, Argentina) compared with the Chagas Rapid First Response (Lemos Laboratories, Argentina) (p = 0.2008); and WL Check Chagas (Wiener Lab, Argentina) compared to ACCU-TELL Chagas Cassette (AccuBiotech Co. Ltd, China); as well as Chagas Rapid First Response (Lemos Laboratories, Argentina) compared to ACCU-TELL Chagas Cassette (AccuBiotech Co. Ltd, China) (p = 0.2008) (Table 3).

### Usability

Qualitative data, relating to the operating and storage requirements of each index test, were obtained from the IFU provided by the manufacturers (Table 1). All RDTs required the same operating temperature (15–30°C), and similar ranges of transport / storage temperatures (1–30°C), and in-use stability range (between 15–35 minutes following the addition of buffer). In terms of sample type, all of the RDTs worked with plasma, serum and whole blood. However, the SD Chagas Ab Rapid only worked whole blood obtained by venipuncture, while the other three tests allow the use of fingerstick blood. The sample volume of whole blood sample volume required ranged between 5 and 100 μl.

The usability score was estimated for each test, by all laboratory technicians (S2 Table). The score was calculated by considering: appearance of the background in the device after testing, test and control band intensity, quality of package insert, ease of reading, sample dispenser included in the kit, lancet included in the kit. The laboratory technicians all assigned a usability score higher than the average [11] for all RDTs.

## Discussion

To reduce and ultimately eliminate CD as a public health problem it is necessary to increase diagnostic coverage [26]. In this context, although RDTs are still only recommended for screening, and more evidence of its diagnostic utility is needed, RDTs have emerged as an option for the rapid and conclusive diagnosis of *T*. *cruzi* infection, assisting provision of treatment, improved adherence and contributing to the prevention of vertical transmission [16]. In our study, the four RDTs evaluated each displayed very good performance, both in terms of sensitivity and specificity, with similar values to those reported previously for these tests [13,19,26].

The SD Chagas Ab Rapid by Standard Diagnostic (Korea), WL Check Chagas by Wiener Lab (Argentina), and ACCU-TELL Chagas Cassette by AccuBiotech Co. Ltd (China) showed high sensitivity. These tests were able to correctly classify more than 196 out of 200 infected samples, individually tested by each RDT. The Chagas Rapid First Response by Lemos Laboratories (Argentina), WL Check Chagas by Wiener Lab (Argentina), and ACCU-TELL Chagas Cassette by AccuBiotech Co. Ltd (China) showed high specificity. These tests were able to correctly classify between 186 and 192 out of 200 non-infected samples, individually tested by each RDT.

In our study, Cohen’s *Kappa* for the majority of the RDTs showed an almost perfect concordance with the reference standard, except for the SD Chagas Ab Rapid by Standard Diagnostic (Korea), that showed a substantial concordance given that this test displayed a higher proportion of false-positive results compared with the other tests. However, none of the RDTs had statistically significant different overall agreement with the reference standard (*Kappa* index ≥ 0.8). Our findings are in agreement with the increasing recent evidence suggesting that the performance of RDTs is comparable to that of laboratory-based tests [18,27,28].

Interestingly, in another laboratory evaluation with a similar study design to our study [18], sponsored by FIND, the authors assessed WL Check Chagas by Wiener Lab (Argentina), SD Chagas Ab Rapid by Standard Diagnostic (Korea), and Chagas Rapid First Response by Lemos Laboratories (Argentina), using samples from Colombian individuals and the diagnostic algorithm in Colombia as reference. These RDTs displayed lower sensitivity (94%; 86.7% and 81.8%, respectively) and higher specificity (98.9%; 99.6% and 98.6%, respectively) compared to our study. Also, the diagnostic performance of the tests could be influenced by other factors such as regional differences in parasite antigenicity due to different DTUs.

In terms of usability, all of the RDTs we evaluated required the same operating conditions. Regarding sample type, only the SD Chagas Ab Rapid by Standard Diagnostic (Korea) requires whole blood obtained by venipuncture rather than fingerstick and a larger sample volume (0.1 ml), more than twice the volume required for the other tests. This might limit this test’s potential usefulness under real-world conditions with capillary fingerstick samples. According to the users, all of the tests were easy to handle and interpret (S2 Table).

Chagas disease, along with human immunodeficiency virus (HIV), syphilis, and hepatitis B virus, is included in the Framework for Elimination of Mother-to-Child Transmission (EMTCT Plus) initiative of the PAHO member states. The use of RDTs for HIV and syphilis would allow treatment and care to be initiated with no unnecessary delays, favoring immediate adherence, and reducing the risk of transmission [8]. Screening with RDTs is currently used for the other three diseases in the EMTCT Plus framework, but not yet for CD. In light of the results obtained by Eguez *et*. *al*. (2017) (with Chagas Stat-Pak, by Chembio, and Chagas Detect Plus, by Inbios) [16] and Lopez Albizu *et*. *al*. (2020) (with SD Chagas Ab Rapid by Standard Diagnostic and WL Check Chagas by Wiener Lab) [21], and in our study, with any of the RDTs that work with a small volume of serum as a sample, RDTs are comparable to the reference laboratory-based tests, and could potentially be used not only as screening tests but also as a secondary confirmatory tool, similar to what is already the case for diagnosing HIV infections in perinatal care centers. Together, these results show that RDTs on their own are not sufficient as stand-alone tests, but they could be particularly useful in remote areas that lack basic equipment, such as a centrifuge, where it is difficult to follow recommendations for diagnosis [27], but further evidence about performance and economic impact in different real clinical scenarios is further needed.

Truyens *et*. *al*. (2021) have demonstrated that a combination of two RDTs showed reactivity very similar to that of the combination of two ELISAs, based on a cohort of *T*. *cruzi-*infected women and including blood/plasma samples from Argentina, Honduras, and Mexico, thus confirming the usefulness of RDTs for screening [29]. This strategy could potentially also reduce healthcare costs, given that RDTs are considered to be a cost-effective strategy compared with laboratory-based diagnostic methods, if the logistical costs are included, such as the cost of facilities and of training personnel in the handling of samples and interpretation of the results [16].

Although many RDTs for CD have been described, not all are available for purchase by health structures in CD-endemic countries, so it is important that from the various RDTs each performs well. Our initial objective was to compare seven RDTs, but this was not possible because all were not all available for procurement in Argentina.

It will be necessary to confirm the optimal performance of the RDTs we evaluated in a field evaluation study using capillary fingerstick samples, along with the analysis of usability and cost-effectiveness, to be able to recommend their use in different scenarios and with different sample types. However, given the sensitivity and specificity values obtained under controlled laboratory conditions in our study, our hypothesis is that the use of RDTs could strengthen local health structures. The accuracies of all RDTs evaluated in this study, conducted by the national reference laboratory for diagnosis of CD, are considered sufficiently good to recommend their use in Argentina to increase diagnosis rates at the primary healthcare level and reduce the time to diagnosis, once their optimal performance has been demonstrated in real-world settings.

## Conclusion

In conclusion, our study demonstrates that, under controlled laboratory conditions, commercially available RDTs for CD have a performance comparable to the Argentinian diagnostic algorithm, which is based on laboratory-based serological tests, including ELISA, IHA, and IIF. The current low rates of diagnosis of CD, due to the complex diagnostic process involved, represent the first key barrier that must be overcome to improve healthcare for patients with *T*. *cruzi* infection, who frequently need to travel to secondary or tertiary healthcare centers far from their homes for a test and then wait several weeks to obtain a diagnosis and access treatment and care. Rapid tests could modernize and simplify the diagnostic process for patients and healthcare providers alike, potentially providing a confirmed diagnosis result at the primary healthcare level. However, further studies must be conducted to evaluate the performance of RDTs using fingerstick blood samples in real-world conditions at primary healthcare centers.

## Supporting information

S1 Table Number of positive/negative samples needed for a range of estimated sensitivities/specificities. (DOCX)

S2 Table Usability scores of the RDTs evaluated by four operators. (DOCX)

S1 Fig Images of each index test verified with the WHO anti-*Trypanosoma cruzi* I and II antibody reference panel (NIBSC). a) WL Check Chagas, positive control result; b) WL Check Chagas, negative control result; c) Chagas Rapid First Response, positive control result d) Chagas Rapid First Response, negative control result; e) ACCU-TELL Chagas Cassette, positive control result; f) ACCU-TELL Chagas Cassette, negative control result; g) SD Chagas Ab Rapid, positive control result; h) SD Chagas Ab Rapid, negative control result.
(TIF)

S1 Data Excel spreadsheet containing reference test results. (XLSX)
