# Supplementary material for: Comparative evaluation of four rapid diagnostic tests that detect human Trypanosoma cruzi-specific antibodies to support diagnosis of Chagas Disease in urban population of Argentina
Source: PLoS Negl Trop Dis. 2024 Mar 15;18(3):e0011997. doi: 10.1371/journal.pntd.0011997 (PMC10971758; doi:10.1371/journal.pntd.0011997)
Supplement: S1 Table — (DOCX) [file pntd.0011997.s001.docx]

**S1 Table.** Number of positive/negative samples needed for a range of estimated sensitivities/specificities.

| **Estimated sensitivity/specificity** | **95% confidence interval** | **Number of positive / negative samples needed** | **Number of positive/negative samples considering 30% of potentially poor-quality specimens** |
| --- | --- | --- | --- |
| 97.5% | ±2.5% | ≥149.8 | 200 |
| 95.0% | ±3.5% | ≥149.8 | 200 |
| 90.0% | ±4.8% | ≥150.1 | 200 |
| 85.0% | ±5.7% | ≥150.7 | 200 |
