# Supplementary material for: Comparative evaluation of four rapid diagnostic tests that detect human Trypanosoma cruzi-specific antibodies to support diagnosis of Chagas Disease in urban population of Argentina
Source: PLoS Negl Trop Dis. 2024 Mar 15;18(3):e0011997. doi: 10.1371/journal.pntd.0011997 (PMC10971758; doi:10.1371/journal.pntd.0011997)
Supplement: S2 Table — (DOCX) [file pntd.0011997.s002.docx]

**S2 Table.** Usability scores of the RDTs evaluated by four operators

| **Test** | **Appearance of the background in the device after testing** | **T/C band intensity** | **Quality of package insert (Instructions For Use)** | **Ease of reading** | **Sample dispenser included in the kit** | **Lancet included in the kit** | **Score** |
| --- | --- | --- | --- | --- | --- | --- | --- |
|  | **Clear (2) Dark (1)** | **Frequently intense (2)  Frequently weak (1)** | **Very good (3)  Good (2)  Fair (1)** | **Effortless (3)  Difficult (2)  Very difficult (1)** | **Yes (2)  No (1)** | **Yes (2)  No (1)** |  |
| **WL Check Chagas** | 2.0 | 2.0 | 2.3 | 3.0 | 1.0 | 1.0 | **11.3** |
| **SD Chagas Ab Rapid** | 2.0 | 1.8 | 2.3 | 3.0 | 1.0 | 1.0 | **11.1** |
| **Chagas Rapid First Response** | 2.0 | 2.0 | 2.3 | 2.8 | 2.0 | 2.0 | **13.1** |
| **ACCU-TELL Chagas Cassette** | 2.0 | 2.0 | 2.3 | 2.8 | 2.0 | 1.0 | **12.1** |
