# Supplementary figures and images for: Comparative evaluation of four rapid diagnostic tests that detect human Trypanosoma cruzi-specific antibodies to support diagnosis of Chagas Disease in urban population of Argentina
Source: PLoS Negl Trop Dis. 2024 Mar 15;18(3):e0011997. doi: 10.1371/journal.pntd.0011997 (PMC10971758; doi:10.1371/journal.pntd.0011997)

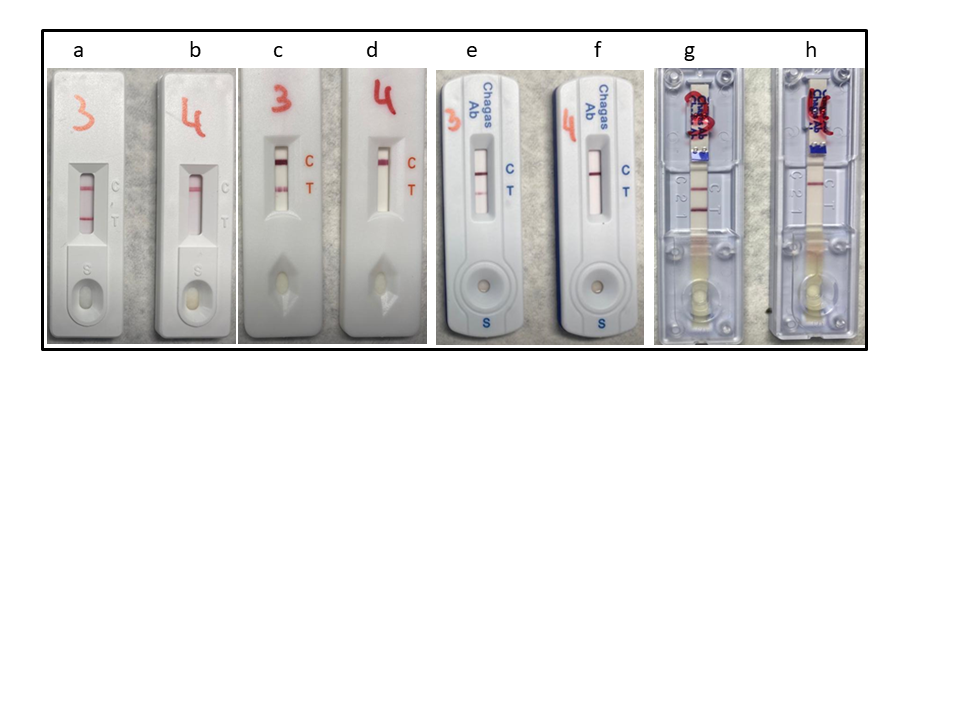

Supplement: S1 Fig — a) WL Check Chagas, positive control result; b) WL Check Chagas, negative control result; c) Chagas Rapid First Response, positive control result d) Chagas Rapid First Response, negative control result; e) ACCU-TELL Chagas Cassette, positive control result; f) ACCU-TELL Chagas Cassette, negative control result; g) SD Chagas Ab Rapid, positive control result; h) SD Chagas Ab Rapid, negative control result. (TIF) [file pntd.0011997.s003.tif]
